# The Functional Role of Polyphenols Across the Human Lifespan

**DOI:** 10.3390/ijms262211074

**Published:** 2025-11-16

**Authors:** Bekir Enes Koca, Sümeyye Sarıtaş, Mikhael Bechelany, Sercan Karav

**Affiliations:** 1 Department of Molecular Biology and Genetics, Çanakkale Onsekiz Mart University, Çanakkale 17000, Türkiye; 2 Institut Européen des Membranes (IEM), Unité Mixte de Recherche (UMR) 5635, University of Montpellier, ENSCM, CNRS, F-34095 Montpellier, France

**Keywords:** life stages, age-specific effects, infancy, childhood, adulthood, aging, secondary metabolites

## Abstract

Polyphenols are bioactive molecules that occur naturally in plants and exhibit a diverse array of properties, including antioxidant, anti-inflammatory, and anti-obesity effects, all of which have been supported by numerous studies. They are categorized into four main groups: flavonoids, phenolic acids, stilbenes, and lignans. Polyphenols demonstrate a wide range of health-promoting effects throughout human life, from the womb to old age. They can exert these effects by modulating signaling pathways, regulating gut microbiota, influencing gene expression, and regulating epigenetic pathways. This comprehensive review summarizes the evidence regarding polyphenol intake across various life stages, exploring their effects on immune function, cognitive development, cardiovascular health, and healthy aging. These findings highlight the potential role of polyphenol supplementation in supporting lifelong health. It also emphasizes the significant impact of polyphenols on mental health issues and obesity, which have become more prevalent in modern life. The review also highlights the distinct requirements for each age group, due to changes in metabolic and cellular functions, as well as the age-specific effects of polyphenols. Recent in vitro, in vivo and clinical studies were reviewed to evaluate the biological effects of polyphenols. In the current literature, there are limited studies that directly compare the effects of polyphenols specific to different life stages and comprehensively address the results. This review aims to provide a framework to guide future research by evaluating the effects of polyphenols used in early life, adulthood, and old age.

## 1. Introduction

**Figure 1:**
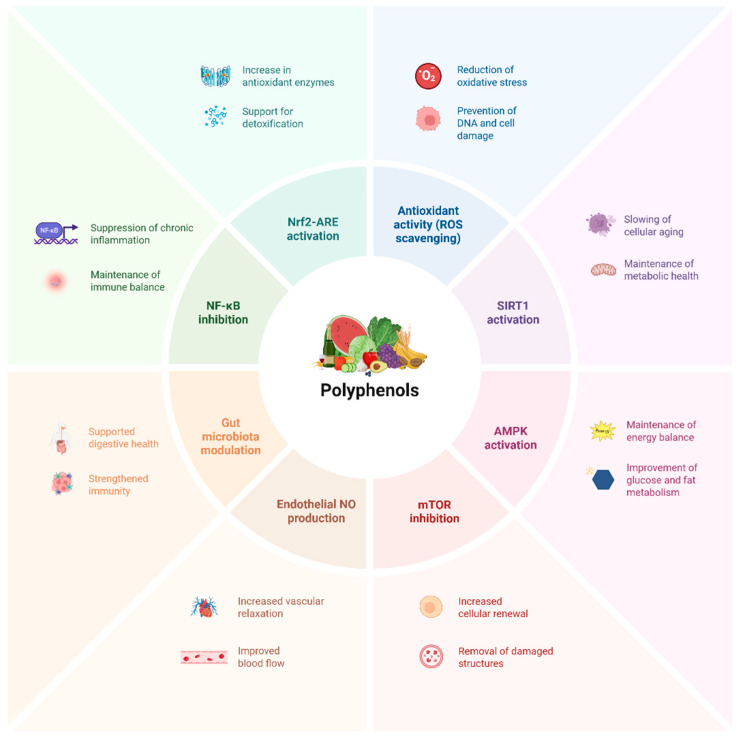
Primary mechanisms illustrating the biological effects of polyphenols.

Polyphenols are a group of biologically active compounds synthesized as secondary metabolites in plants and possessing various structural characteristics. These compounds exert diverse effects on numerous physiological processes and cellular functions within the organism [1]. Various studies have demonstrated that polyphenol-containing substances have anti-inflammatory, antithrombogenic, anti-allergenic, anti-diabetic, anti-cancer, and antioxidant properties, among other health-promoting and preventive advantages [2,3]. Due to their various biological effects, polyphenols are considered protective compounds in the prevention of chronic and degenerative diseases. According to Zhang et. al. polyphenols can help prevent cancer, diabetes, liver disease, neurological disorders, and cardiovascular diseases [4]. The structure of these molecules typically features one or more hydroxyl groups bonded to one or more phenyl rings [5]. According to their molecular structure, these substances can be divided into four main subclasses: lignans, stilbenes, phenolic acids, and flavonoids [6]. Flavonoids are the primary and most extensively researched category of polyphenols, encompassing several subclasses such as flavones, flavoles, anthocyanins, isoflavones, chalcones, dihydroflavols, dihydroflavones, proanthocyanidins, and flavonoid carbosides. Leaves, roots, stems, and flowers in plants can produce flavonoids. They are found in high amounts in fruits, particularly apples and plums. Among vegetables, onions and broccoli are notable sources. They are also commonly found in citrus fruits [7]. Another significant subgroup of polyphenols is phenolic acids. Phenolic acids are concentrated particularly in whole grains, coffee, and various types of fruits and vegetables. These compounds, which are of scientific interest due to their various biological effects, exhibit antidiabetic, anti-inflammatory, and potent antioxidant properties (Figure 1). Structurally, they are classified into two main groups: hydroxybenzoic acids (e.g., gallic acid, protocatechuic acid) and hydroxycinnamic acids (e.g., ferulic acid, caffeic acid) [1]. Stilbenes are also a separate subclass of polyphenols. Resveratrol, the most researched compound in this group, stands out for its potential effects in preventing or treating cardiovascular diseases, as well as cancer and neurodegenerative disorders [8,9]. It is found in nature, particularly in fruits such as grapes and strawberries. However, its high concentration in red wine has led to intense debate in scientific circles about the potential health benefits of resveratrol. In addition, lignans found in different plant varieties have been shown to exhibit anti-aging, diabetes-preventing, antibacterial, and antiviral effects. Like other polyphenol classes, lignans also exhibit hepatoprotective, immunomodulatory, cardioprotective, and neuroprotective properties [10].

Polyphenols have been extensively studied because of their potential uses in the prevention and treatment of disease, and they are well known for their strong anti-inflammatory and antioxidant qualities [11]. Examples of polyphenols with strong anti-inflammatory and antioxidant effects include quercetin and resveratrol, which modulate various cellular stress responses and inflammation-related regulatory molecular pathways, such as AMP-activated protein kinase/sirtuin (AMPK/SIRT) and extracellular signal-regulated kinase 1/2–ribosomal S6 kinase–neuronal nitric oxide synthase (ERK1/2-RSK-nNOS). Citrus peels also contain hesperidin, a flavonoid that has been associated with promising therapeutic benefits, including anti-inflammatory and anti-atherosclerotic. These well-established impacts on cellular functions clarify the diverse biological activities of polyphenols, indicating their potential preventive roles in areas such as diabetes management and protection of cardiovascular and brain health [12,13].

In biological systems, polyphenols exert multifaceted effects on health by regulating different cellular signaling networks and gene expression mechanisms. Apigenin, quercetin, kaempferol, curcumin, resveratrol, genistein, and gallic acid are notable examples of dietary phenolic compounds [13,14]. These compounds can have an effect on different cellular signaling networks. In studies related to chronic diseases, they have been reported to have both preventive and therapeutic potential. This effect is considered to be connected to the regulation of inflammatory processes and cellular life pathways. Additionally, they exhibit effects on microRNAs, caspases, Bcl-2 family proteins, and fundamental signaling cascades like Wnt/β-catenin, NF-κB, MAPK, and PI3K/Akt. It has been demonstrated that the interaction between polyphenols and gut microbiota enhances the diversity and richness of gut microbial communities [15]. Short-chain fatty acids (SCFAs), trimethylamine N-oxide, dopamine, bile acids, and lipopolysaccharides are among the gut microbiota metabolites that polyphenols affect. These metabolites are essential for the control of gut metabolism. Additionally, polyphenols can be processed by the gut flora, changing their bioavailability and biological activity [16].

These compounds have well-known anti-inflammatory and antioxidant properties, but they can also affect gene expression, control intracellular signaling pathways, and influence epigenetic processes [17]. Through the modulation of many signaling pathways, resveratrol, for instance, can significantly reduce inflammation and ameliorate Parkinson’s disease (PD), a neurodegenerative disorder [18]. In another example, it was observed that polyphenol-rich lingonberry (*Vaccinium vitis-idaea* L.) reduced adverse metabolic changes by affecting gene expression in a mouse model [19]. The third instance worth noting is the potential of polyphenol-containing foods to modulate telomere activity by influencing epigenetic pathways, thus affecting cellular aging and health span [20]. Polyphenols delay cellular aging by reducing oxidative stress and preserving telomere stability. These effects are related to molecular mechanisms such as the epigenetic upregulation of hTERT (human Telomerase Reverse Transcriptase) gene expression via the NAD^+^-dependent deacetylase sirtuin 1 (SIRT1) and the support of mitochondrial biogenesis through the activation of the energy sensor AMP-activated Protein Kinase (AMPK).

Numerous studies on polyphenols generally focus on a specific age group or disease model. The effects of polyphenols throughout various life stages are often investigated separately. However, from the prenatal period to old age, people’s physiological needs, metabolic priorities, and environmental responses change. This change causes the biological effects of polyphenols to manifest in different ways over time. This review aims to synthesize the existing literature on how polyphenols affect different physiological systems according to life stages from a period-based perspective. Thus, the similarities, differences, and common biological outcomes of polyphenol effects are comprehensively evaluated, starting from pregnancy and early development through childhood, adulthood, and old age. This approach systematically analyzes the age-based effects of polyphenols by integrating fragmented information in the literature. Moreover, this period-based assessment provides an important conceptual foundation that will contribute to the consideration of age-specific physiological requirements and the development of personalized nutrition strategies in the future use of polyphenols as dietary supplements or functional ingredients.

**Figure 2:**
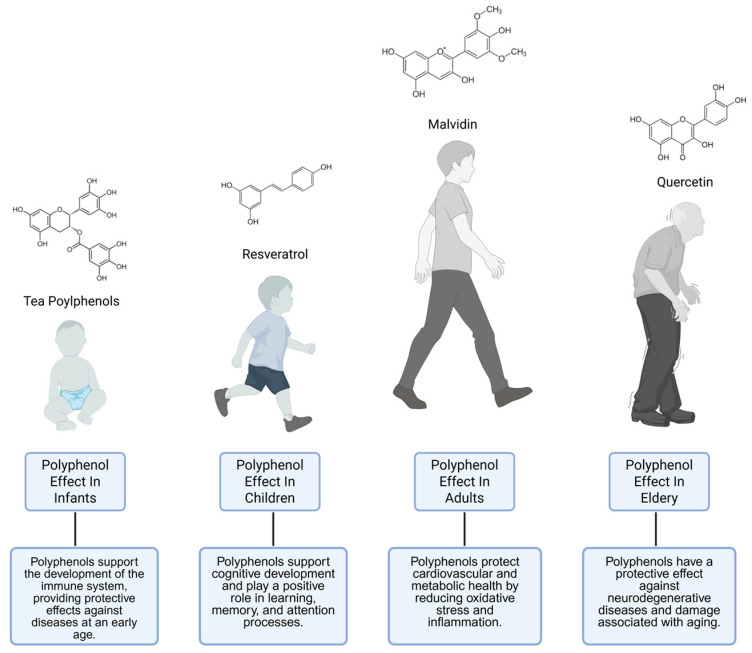
Effects of polyphenols at various stages of human life: infancy, childhood, adulthood, and elderly. Note: The effects illustrated are not exclusive to each life stage; rather, they may occur throughout the lifespan, although some effects tend to be more prominent at specific stages.

This review article discusses the potential effects of polyphenols throughout different stages of life, starting from the womb and continuing through infancy, childhood, adulthood, and old age. Particularly, the potential benefits of these effects, specific to each life stage and the biological mechanisms through which they arise, are examined. Additionally, the role of polyphenols in health is examined in relation to age-related changes in metabolic status. In this context, the article aims to present the potential contributions of lifelong polyphenol consumption from a holistic perspective. Because metabolic and cellular functions evolve throughout life, each age group has distinct needs. It is thought that the effects of polyphenols, which offer a variety of benefits, may differ with age (Figure 2). Moreover, this period-based assessment provides an important conceptual foundation that will contribute to the consideration of age-specific physiological requirements and the development of personalized nutrition strategies for the future use of polyphenols as dietary supplements or functional ingredients. They play a role in maternal obesity and cardiometabolic disorders, positively influencing the fetus during the early stages of life and supporting microbiome development in infants [21,22]. This occurs through the transfer of substances via the placenta and through breast milk after birth. Additionally, contributing to neurological and cognitive development in childhood and adolescence [23]. Animal, human, and public health studies indicate that it has a range of effects, including enhancing cardiovascular endurance in both adulthood and old age, regulating metabolic balance, and promoting emotional and cognitive resilience as well [24]. They also have significant effects on aging processes [25]. Due to their antioxidant and cell-protective properties, they are reported to have the potential to extend lifespan and delay aging. Regular consumption of polyphenol sources obtained from the Mediterranean and Japanese diets may support longevity by reducing the risk of chronic diseases.

## 2. Polyphenols in Early Life (Infancy to Adolescence)

Numerous studies have researched the use and effects of polyphenols in adults; however, the timing of exposure concerning the stage of biological development may influence the effects of polyphenols. Therefore, there is a crucial need to investigate early polyphenol intake to uncover age-related differences in the potential effects of polyphenols during early development [26].

### 2.1. Prenatal and Lactational Polyphenol Exposure

Polyphenols have many positive effects via the placenta on the fetus, in infants after birth through breast milk, and later in childhood and adolescence. They meet period-appropriate needs, including immune development in infancy, cognitive development, and behavioral effects in childhood, also neuroprotective effects in adolescence [22,23]. These early health-promoting effects may contribute to long-term physiological and cognitive development [27].

It is being investigated whether the experience in the womb may have an impact on health status and disease risks later in life after birth [28]. Polyphenols ingested during pregnancy can be transmitted to the fetus through the placenta and may also affect the offspring paternally. In a study, it was observed that fruit and vegetable intake beneficially affected the quality of sperm in male rats [29]. In a recent study by Pires et al., the maternal and paternal effects of polyphenols (cyanidin, quercetin, and ellagitannins) contained in blackberries were investigated in C57BL6 mice [26]. Fathers who consumed a polyphenol-rich blackberry extract during the pre-conception phase (BF group) exhibited a higher plasma antioxidant capacity compared to the control fathers. Elevated hepatic superoxide dismutase (SOD) activity in the livers of the fathers in the BF group, with reducedSOD and catalase (CAT) activity in the testicles. No significant change in GPx activity. Fathers in the BF group showed an increased percentage of sperm with normal morphology and the amount of daily sperm production. Paternal subjects treated with methanolic blackberry extract exhibited a tendency toward improved pregnancy rates (*p* = 0.067). Women who consume blackberries have decreased liver SOD activity compared to control mothers. All forms of parental treatment (paternal, maternal, or combined) lower perinatal mortality observed in the offspring. Co-administered parental supplementation of blackberry polyphenol consumption markedly reduced perinatal mortality and increased the plasma antioxidant capacity of juvenile females.

Additionally, polyphenol consumption during pregnancy and lactation may impact milk composition and functional properties, with potential downstream effects on neonatal immune function and brain maturation [22,28,30]. A study has shown that maternal dietary polyphenols can enhance the amount and quality of lipids (triacylglycerols and cholesterol) found in breast milk [31]. These lipids are vital nutrients that play a vital role in the healthy growth and development of infants. Another investigation found that maternal consumption of polyphenols during lactation is related to a decreased risk of early cessation of breastfeeding [32]. Supplementing mothers with cranberries improves the antioxidative status of breast milk, strengthens the antioxidant system, and thus may provide greater protection for the breastfed infant.

In conclusion, there are very few studies on this topic, and more experiments need to be conducted to learn about the potential impacts of polyphenols in early life.

### 2.2. Polyphenol Effects in Infancy

The numerous effects of polyphenols on the development of cognitive and immune functions highlight the potential of these compounds during critical years of growth [33,34]. The postnatal stage is recognized as a sensitive period when the immune system begins to take shape under various environmental influences [35]. Polyphenols can support the maturation of the immune system during early developmental stages. They exert this effect through multiple pathways, including regulating cytokine production, enhancing antioxidant defense, and modulating immune cell activity [36,37,38].

A study conducted by Yang et al. indicates that polyphenolic compounds increase total antioxidant activity and may contribute to protective effects against inflammation by altering macrophage polarization in an anti-inflammatory direction, thereby strengthening immune function [27]. In a research study on largemouth bass, dietary TPs strengthened immune responses by modulating the expression of relevant immune genes. By promoting homeostasis in the gut microbiota, polyphenols have also been shown to enhance gut health, and this is important for immunity. The effects of tea polyphenols on the immune system, antioxidant capacity, and intestinal microbiota of weaned goat offspring have been investigated in a different parallel study [39]. According to a study, weaned goat kids may have compromised immune function, which increases their risk of illness. In goat kids that received tea polyphenol supplementation, immune defense was enhanced, and inflammatory damage was diminished. This improvement was correlated with a marked reduction in the expression of TLR4, MyD88, and NFκB genes. In addition, a dietary supplement of 4 g/kg of tea polyphenols regulated the intestinal microbiota and improved immune function through its antioxidant effects. The combination of tea polyphenols and antibiotic supplementation exhibited a suppressive effect on the proinflammatory cytokine profile, which indicates inflammation in the duodenum, jejunum, and ileum, specifically involving cytokines like IL-6, IL-1β, TNF-α, and IFN-γ. Furthermore, it caused a notable boost in anti-inflammatory cytokine levels (IL-10). By targeting and reducing the activity of the TLR4/MyD88/NFκB pathway and suppressing the expression of cytokine-signaled genes, it can improve the immune functions of the intestinal epithelial barrier and decrease the impairment induced by proinflammatory processes. Moreover, it has a regulatory effect on the antioxidant capacities of goat kids by regulating nitric oxide (NO) production.

In another study, Wen et al. investigated the effects of hydroxytyrosol on piglets under oxidative stress induced by diquat [40]. The epithelial cells and the mucosal protective layer that comprise the intestinal barrier serve as a defense against pathogens through tight junction proteins and the mucus layer. However, oxidative stress inducers like diquat can disrupt this structure, leading to a reduction in villus height, a weakening of tight junctions, and an increase in intestinal permeability. Following diquat administration to piglets, hydroxytyrosol treatment was observed to reduce oxidative stress.

Additionally, preclinical studies on rodent animal models have suggested that polyphenol consumption during infancy has the potential to improve cognitive functions such as learning, memory, and motor coordination and reduce anxiety [41]. Resveratrol and piceatannol, in particular, have been reported to enhance both memory and overall cognitive function. The neonatal period, however, is indicated by a strong vulnerability to hypoxia and ischemia. Research shows that when polyphenols are given before such hypoxic-ischemic episodes, their neuroprotective effects appear stronger. These benefits are thought to arise not only from their antioxidant and anti-inflammatory capacity but also from their ability to modulate key cellular and molecular processes within the brain.

### 2.3. Polyphenol Effects in Childhood

Chemosensory responses and the maturation of cognitive abilities, including learning and memory, are especially important during infancy and childhood. Evidence suggests that polyphenols may provide additional support for these developmental processes [42,43].

One investigation, for instance, assessed how a combination of inulin and blueberry-derived polyphenols influenced cognitive performance measures in children [43]. Executive functions (EF) encompass the cognitive processes that evolve and mature from childhood through adolescence and into early adulthood. These executive functions are regulated by the frontal regions of the brain. Supporting healthy cognitive development during the neonatal and infant stages is crucial because dysfunctions in the frontal lobe can have lasting effects while neurological development is still in progress. The developmental stage spanning 7 to 10 years is a crucial stage of development, marked by the growth of the brain’s frontal lobes. Consequently, child participants in this age range were chosen for the study, and it was hypothesized that gut-microbial interventions could enhance EF and overall development [43]. Significant improvements in delayed recall memory performance and vocabulary learning (final acquisition) after supplementation. Blueberry intake, high in flavonoids, improved attention, cognitive inhibition, visual-spatial memory, and executive functions in 8- to 10-year-old children within 2–6 h after consumption. Regular dietary intake led to improvements in long-term memory function. Additionally, the consumption of polyphenol-rich blueberries influenced gut microbiota, leading to a notable increase in Bacteroidetes, which are linked to enhanced cognitive, language, and motor development scores. The findings of this study indicate that foods such as inulin and polyphenol-rich blueberries can significantly affect cognitive performance and memory.

Resveratrol has been observed to have multifaceted effects on children’s health at both the local and systemic levels [44,45,46]. However, it is understood that these effects vary depending on the formulation, dosage, and model type used. A randomized clinical trial conducted in early childhood observed that cyclodextrin-based RES-HPβCD spray significantly reduced dental plaque and gingivitis and increased saliva pH toward neutral values [45]. It was also reported that the application showed no adverse effects, was well tolerated by children, and increased local bioavailability. In another study, the impact of RES was investigated in Osteosarcoma (OS) cell lines, a common bone sarcoma in pediatric patients [44]. It was shown that RES has selective anti-cancer potential, inhibiting tumor cell proliferation at high doses without affecting healthy cells. However, this model is at the preclinical level and has limited applicability to systemic dosing and metabolic stability in the human body. Another study investigated formulations for children affected by liver disease [46]. It was demonstrated that encapsulating RES in TPGS micelles, a derivative of vitamin E, increased its solubility by up to 15 times, preserved its antioxidant activity, and improved its potential systemic bioavailability. These three approaches demonstrate the pharmacological potential of RES on distinct levels. These observations suggest that RES may possess a multifaceted effect profile in biological processes specific to childhood and that these effects justify more comprehensive evaluation.

Another study examined polyphenol intake and its effects in children with cancer [47]. There is some proof that polyphenol consumption is low in childhood cancer patients. Children with cancer had an average daily polyphenol intake of 173.31 ± 141.02 mg and were observed to have a lower level compared to children without cancer. In addition, an increase in consumption of polyphenols was observed with increasing age of the patient in the study: 1–3 years: 100.20 mg/day; 4–11 years: 153.44 mg/day; 12–18 years: 273.28 mg/day. The study emphasized that improving nutritional intake by focusing on polyphenols may have a supportive effect in suppressing treatment-related side effects, as treatment damages the body’s antioxidant system. Serious studies evaluating the intake or effectiveness of polyphenol-rich diets in childhood cancer patients are still limited. In this study, which represents one of the initial investigations into this topic, the impact of polyphenol intake was assessed in 59 pediatric cancer patients. However, the generalizability of the findings is quite limited. It is stated that it may be valuable in nutritional interventions for childhood cancer patients in the future.

In another study, researchers examined the methanolic extract of Eleutherococcus divaricatus fruits using serum samples from children diagnosed with acute leukemia (AL) [48]. Chlorogenic acid, protocatechuic acid, and eleutheroside E polyphenols were detected in these fruits. The extract showed a very high inhibition activity against human serum hyaluronidase obtained from children with an average age of 7 before starting treatment. This inhibition activity ranged from 76% to 86%. The study suggests that polyphenolic compounds may be responsible for this effect. Polyphenol therapy may be a new strategy in the treatment of inflammatory diseases such as leukemia. However, it is important to note that the findings may vary due to differences among individual patients. These observations indicate that potential synergistic or antagonistic interactions with current therapeutic agents should be reassessed in larger patient populations. Several studies addressing early-life polyphenol intake across various research models are summarized (Table 1).

## 3. Adulthood: Metabolic Health and Disease Prevention

Polyphenols, especially with their antioxidant and anti-inflammatory effects, can support cardiovascular health, helping to prevent hypertension and other cardiovascular health problems [52]. Polyphenols may also contribute to modulating lipid metabolism disorders by exerting anti-obesity effects [53]. Additionally, they demonstrate properties that help combat stress and related disorders affecting many people worldwide [54].

### 3.1. Cardiovascular Protection

Cardiovascular diseases are chronic conditions that negatively affect the heart and circulatory system [55]. These are some of the world’s leading causes of death. Various factors contribute to the progression of cardiovascular diseases. In addition to factors like genetic inheritance, gender, age, and lifestyle choices such as smoking, physical activity, and diet also impact the cardiovascular system. Among these factors, diet significantly impacts the management of cardiovascular disease and helps control risk factors like diabetes, obesity, and hypertension.

The Mediterranean diet is abundant in polyphenols and is recognized for its beneficial effects on cardiovascular health, which is an example of the heart-protective properties of polyphenols [56]. The Mediterranean diet is based on the consumption of fruits and vegetables and contains several bioactive compounds, including polyphenols. Studies have exhibited that polyphenol-rich diets have preventive benefits against many chronic diseases. Polyphenols play a crucial role in maintaining good health, notably in preventing cardiovascular diseases. They improve the function of the heart and blood vessel linings, increase high-density lipoprotein (HDL) cholesterol, decrease low-density lipoprotein (LDL) cholesterol, promote antiplatelet and anti-inflammatory effects, and help reduce systemic inflammation and lipid profiles. In particular, polyphenols, for instance, trans-resveratrol and quercetin, have been shown to be associated with better cardiovascular health [57].

According to a recent study, polyphenols obtained from white grape residues regulated the oxidative stress response of blood pressure and also modulated inflammatory mediator levels in an experimental hypertension model induced by dexamethasone [58]. Polyphenols modulate inflammatory mediator levels as well as regulate blood pressure and oxidative stress response in an experimental model of hypertension stimulated by dexamethasone, according to a new study evaluating the effects of polyphenols extracted from white grape residues. Dexamethasone (DEXA) administration to rats increased systolic (SBP), diastolic (DBP), and mean blood pressure (MBP). In addition, it is increased reactive oxygen species (ROS) production, reduced nitric oxide (NO) bioavailability, and caused hypertension by suppressing NO synthase gene expression. DEXA administration also increased total oxidative stress (TOS), oxidative stress index (OSI), and malondialdehyde (MDA) levels, while decreasing total antioxidant capacity (TAC), NO, and total thiol (THIOL) levels. High-dose polyphenol extract (795 mg/kg) administration significantly reduced SBP and prevented increases in TOS, OSI, and MDA. It also significantly increased TAC, NO, and THIOL levels. Lower-dose administration (397.5 mg/kg) slightly reduced MDA levels and partially increased NO levels. Lisinopril regulated blood pressure and some oxidative stress parameters when used for comparison; however, it did not significantly affect NO and THIOL levels. These findings demonstrate that grape polyphenols have potent regulatory effects on both blood pressure and oxidative stress responses in experimental hypertension models and may offer certain advantages over conventional antihypertensive treatments.

The potential effects of black chokeberry (*Aronia melanocarpa*) polyphenols on the cardiovascular system have been investigated at various levels, both experimentally and clinically [59,60]. In a study conducted under ex vivo conditions, black chokeberry extracts were reported to exhibit significant vasculoprotective and antioxidant effects on rat aorta [60]. These effects were particularly pronounced under cardiometabolic stressors such as angiotensin II (Ang2), lipopolysaccharide (LPS), and high glucose; extract application significantly reduced oxidative stress markers and improved endothelium-dependent relaxation capacity. Mechanistically, this effect has been associated with catalase-like free radical scavenging activity at high doses (≥100 µg/mL). The study observed no toxic effects up to 500 µg/mL, thus demonstrating a safe antioxidant profile at the tissue level. However, due to the ex vivo nature of the research, bioavailability processes were not evaluated. Additionally, a systematic review and meta-analysis of randomized controlled trials conducted on a clinical scale evaluated the effect of blackcurrant supplementation on cardiometabolic parameters [59]. The result revealed that this supplement did not show a significant and consistent therapeutic effect. Overall results revealed limited changes in indicators such as blood pressure, lipid profile, and glycemic control, while a decrease in systolic blood pressure was observed when the anthocyanin dose exceeded 50 mg/day, and a decrease in low-density lipoprotein cholesterol (LDL-C) and total cholesterol (TC) levels was observed in individuals with low total cholesterol (TC < 200 mg/dL) levels. However, an increase in fasting glucose levels in younger individuals (≤50 years). The meta-analysis emphasized that the findings were of low certainty (GRADE assessment) and that there was a high risk of bias in the studies. When these two studies are evaluated together, although a strong antioxidant and vascular protection potential was demonstrated at the mechanistic level, human clinical data did not fully reflect this effect. This discrepancy has been attributed to the low bioavailability of the specified polyphenols and methodological limitations in human studies. The limited absorption of polyphenols in the gastrointestinal system and the reduced biological activity of their metabolites make it difficult to translate the effect observed in laboratory conditions to the clinical level. In conclusion, current evidence suggests that blackberry polyphenols have the capacity to improve vascular function and reduce oxidative damage in a dose-dependent manner. However, for this effect to reach clinical significance in humans, increased bioavailability and methodologically robust studies are required.

Moreover, another study on soccer players examined the cardiovascular and metabolic benefits of dark chocolate, which contains polyphenols including (−)-epicatechin, (+)-catechin, and proanthocyanidins [61]. Cocoa and dark chocolate may contribute to vasodilation and increased oxygen and glucose delivery. The flavonoids in cocoa improve vascular function by inducing the formation of NO, which improves vasodilation and blood flow. Enhanced blood circulation allows more nutrients and oxygen to reach the muscles, which can speed up the recovery process after exercise and reduce the accumulation of toxic metabolites. According to this pilot study, the antioxidant, anti-inflammatory, and vascular function-enhancing properties of polyphenols, which are found in dark chocolate and cocoa, may help athletes perform better by reducing muscular discomfort. Although direct measurements of blood parameters were not performed, the various benefits provided by polyphenols indirectly suggest positive effects on the cardiovascular system. These antioxidant and vascular effects are consistent with the European Food Safety Authority (EFSA)’s 2010 scientific opinion that flavanol-rich cocoa products can support normal endothelial function [62]. Moreover, in an official document published in 2024, the U.S. Food and Drug Administration (FDA) has approved the use of certain health claims on a conditional basis, based on limited but promising evidence regarding the potential contribution of cocoa flavanols to cardiovascular health [63].

### 3.2. Insulin Sensitivity and Anti-Obesity Effects

As a result of changing living conditions with modern life, such as sedentary life, differences in eating habits, decreased exercise, and irregular sleep, a large proportion of the world population is categorized as overweight or obese [64]. Dietary polyphenols are compounds that show potential for preventing and managing obesity in adults [53].

Because of the overconsumption of foods high in energy in recent years, diseases related to insulin sensitivity, such as type 2 diabetes mellitus (T2DM), obesity, and non-alcoholic fatty liver disease (NAFLD), are extremely common conditions worldwide [65]. Researchers have studied almond shells, which are rich in polyphenol compounds that may lower lipid levels, for their potential to modulate insulin sensitivity and combat obesity. The insulin sensitivity and anti-obesity effects of quercetin, baicalein, and kaempferol, which are the main components of almond skin, were investigated. Baicetin has antioxidative, anti-inflammatory, and antidiabetic effects and is involved in various mechanisms such as inhibition of adipogenesis (fat cell formation), inhibition of carbohydrate hydrolases, and modification of adipocyte differentiation. Kaempferol positively affects glucose homeostasis through the regulation of important cellular signaling pathways like NF-κB, SIRT1, and AMPK. Quercetin, which stands out as a promising agent in the control of metabolic syndrome with its lipid-lowering and anti-inflammatory activity, may be effective on obesity caused by high-fat diets by playing a modulatory role on microbiota and its metabolic products. These polyphenols found in almond hulls exhibited strong binding propensities with important components of cellular signaling, such as mTOR, PI3K, AKT1, STAT3, TNF, and IL6.

According to a study conducted on obese mice fed a high-fat diet, it was observed that the use of polyphenol-rich mulberry (*Morus alba*) branch and leaf extracts (MTE and MLE) reduced obesity and had various health-promoting effects on mice [66]. After supplementation with MTE and MLE, mice exhibited a considerable increase in the mass of brown adipose tissue, which has a regulatory role in glucose and lipid metabolism, along with a reduction in body mass and fat accumulation. It markedly improved fatty liver disease (steatosis) and liver damage and reduced elevated levels of TC, triglycerides (TG), and LDL-C. Several positive effects on insulin sensitivity have also been observed. The supplements improved insulin sensitivity by effectively lowering the insulin resistance index (HOMA-IR) and levels of fasting blood sugar. These effects are thought to be mediated by several mechanisms, including modulation of gut microbiota, reprogramming of SCFAmetabolism, and reduction in inflammation and oxidative stress. MTE and MLE decreased the population of gut bacteria such as Firmicutes, Enterococcus, Leibia, Gemella, and Enterobacteriaceae, which are associated with metabolic disorders in the gut microbiota. It increased the abundance of gut health-promoting bacteria such as Aecalibaculum and Bifidobacterium, thus contributing to a favorable balance in the gut microbiota of obese mice. The conclusion of this experiment is that polyphenol-rich MTE and MLE supplementation interferes with multiple metabolic disorders simultaneously by improving obesity-related metabolic disorders, insulin resistance, and fat accumulation. A study conducted on obese individuals using Kamphaeng Saen mulberry (CMD) showed limited improvements in obesity-related metabolic risk factors [67]. CMD significantly reduced TG levels and inflammation markers such as C-reactive protein (CRP), while maintaining stable fasting plasma glucose levels. However, no significant changes were observed in insulin-related parameters (fasting plasma insulin (FPI) and HOMA-IR). Furthermore, no statistical differences were observed in body composition parameters related to obesity, including body weight, BMI, and fat percentage. These findings of this study indicate that polyphenol-rich MTE and CMD supplements provide similar benefits on obesity-related metabolic disorders, insulin resistance, and fat accumulation, but these effects are more limited in CMD compared to MTE. While both types of mulberry demonstrated beneficial effects in enhancing metabolic disorders and lowering insulin resistance, the impact of CMD was typically less pronounced. However, CMD was observed to have positive effects on certain metabolic markers, particularly lipid metabolism and inflammation.

In summary, polyphenol intake may play an essential role in the protection and treatment of long-term disorders, including diabetes and obesity, through several regulatory pathways [68].

### 3.3. Mood and Mental Health

Depression is generally characterized by a marked drop in mood, loss of enjoyment of daily activities, difficulty focusing, and a persistent feeling of tiredness, and it affects up to 5% of adults in the world population. Polyphenols stand out as potential natural agents that may be effective in coping with stress. They exhibit anti-stress properties in various pathways, including balancing monoamine levels, regulating the gut-microbiota-brain axis, and managing overactivity of the HPA axis [54].

In addition to the potential effects of polyphenols on depression, there is increasing evidence suggesting they may also contribute to stress-related processes [36]. Stress has come to affect the lives of almost every individual in the modern age. Chronic stress or incorrect practices and guidance in stressful situations can have an adverse impact on health. Stress can contribute to a range of medical conditions, such as mental health conditions, metabolic diseases, cardiovascular diseases, and neurodegenerative diseases. Additionally, inflammatory responses that are typically protective can become overactive due to ongoing stress, potentially resulting in chronic inflammation. A study has been performed on the effects of polyphenols on mood and mental health [36]. In this study, the effects of malvidin-3-*O*-glucoside (MG), a compound found in high amounts in grapes, were evaluated in a mouse model exposed to chronic unpredictable stress (CUS). The reluctance to explore new places in mice exposed to CUS has been linked to anxiety, and after MG treatment, the mice showed an increased tendency to explore new areas. This improvement was associated with a reduction in anxiety-like behavior. Moreover, while depressive immobility was observed in the patient mice, activity increased after MG, suggesting that the administered polyphenol may have an alleviating effect on depressive symptoms. MG is an anthocyanin, and by examining the effects of MG ingestion in mice, MG has been demonstrated to play a modulatory role on the stress-induced neuroinflammatory response and has the potential for therapeutic effects on chronic stress, anxiety, and depression.

Consistent with these findings, research on adults examined the consumption of polyphenol-rich beverages among individuals living in the Mediterranean region. The study assessed the possible relationships between this consumption and depressive symptoms, perceived stress levels, and sleep quality [69]. The results obtained indicate that diet may influence mental processes such as mood and sleep patterns. For example, individuals who drank one cup of tea per day were found to have lower levels of depressive symptoms and stress. The study also suggested that polyphenol-rich beverages such as tea, wine, and coffee may exert effects through the gut–brain axis. This mechanism has the possibility of indirectly supporting mental health. Polyphenols enhance the production of SCFAs by altering gut microbiota’s composition to favor beneficial strains, such as *Lactobacillus* spp. and *Bifidobacterium* spp. It has been reported that SCFAs can cross the blood–brain barrier and affect brain functions via neurotransmitter systems (particularly serotonergic and dopaminergic pathways) and the vagus nerve. This mechanism is considered an important pathway that could explain the indirect beneficial effects of polyphenols on stress response, mood, and cognitive response.

Resveratrol can be shown to be an effective polyphenol for mental health. This compound has powerful anti-stress effects as well as anti-inflammatory and free radical protective properties. The effect of resveratrol intake has been studied in various conditions, such as chronic environmental stress, in dogs and mice (Table 2) [70]. As a consequence of different experiments, a decrease in anxiety and worry levels was observed in these animals. In conclusion, resveratrol may support mental health through mechanisms such as regulating the gut microbiota, strengthening immune functions, and reducing oxidative stress.

## 4. Elderly Population: Longevity and Functional Preservation

The prevalence of many chronic diseases rises with aging due to the degeneration of cells and organs. Polyphenols are prominent as potentially effective bioactive molecules against aging-related diseases, age-related skeletal muscle disorders, and life extension (Table 3) [74].

### 4.1. Neurodegeneration Prevention

As people age, their bodies undergo various changes; physiological functions tend to decline, increasing the likelihood of frailty, illness, and mortality. This biological process raises the risk of various diseases, including neurodegenerative illnesses. Old age is a major risk factor predisposing individuals to neurodegenerative disorders [75]. In response to this risk, polyphenols, natural bioactive molecules, exhibit therapeutic effects against neurodegenerative disorders. The therapeutic and preventive effects of polyphenol-containing foods or substances against health problems for instance, Alzheimer’s disease (AD), Parkinson’s disease (PD), Huntington’s disease (HD), multiple sclerosis (MS), dementia, and stroke, which are common in the elderly, are the subject of much research [76]. Moreover, polyphenols act through various mechanisms, including cellular antioxidant systems, neurovascular integrity, proinflammatory signaling cascades, and multifaceted interactions with the gut–brain axis. They are known to play a regulatory role in preventing brain damage caused by excessive neuroinflammation [77].

Inflammation is a significant factor in AD because inflammation is associated with the emergence and worsening of neurodegenerative pathologies. In a study, the use of olive oil (EVOO) polyphenols oleuropein aglycone (OleA) and hydroxytyrosol (HT) was shown to improve markers of neuroinflammation and to have protective benefits against the onset of neural damage [78]. TREM2 controls microglial functions in the brain that play in crucial role in immunity. EVOO polyphenols have been proven effective in activating and destabilizing the TREM2 signaling pathway. They are also active molecules against diseases such as Alzheimer’s with their anti-inflammatory and antioxidant properties. OleA and HT use may be protective for neurodegenerative disorders; these compounds, able to penetrate the blood–brain barrier, may be a potential dietary support against neurodegenerative diseases. Along these lines, health claims approved by the EFSA state that olive oil polyphenols, particularly hydroxytyrosol, may protect against biological damage associated with oxidative stress [79]. Furthermore, the FDA granted GRAS (Generally Recognized as Safe Substance) status in 2015 for the safety of hydroxytyrosol, which supports that this compound is safe for human consumption [80].

A study on AD looked at the therapeutic effect of grape seed polyphenol extract (GSPE) [81]. Amyloid-β (Aβ) plaques and Tau neurofibrils accumulate in the brain and cause Alzheimer’s disease, which is characterized by symptoms related to cognitive decline and memory loss. Due to the challenges and constraints in treating this disease, researchers are exploring new treatment options. Dietary polyphenols may hold therapeutic potential for the prevention of neurodegenerative diseases. In this study, the *Caenorhabditis elegans* (*C. elegans*) model was used, and numerous neuroprotective effects were identified [81]. Additionally, it was observed that the degradation and recycling pattern of damaged proteins and organelles by the autophagy system was altered. Similar results were confirmed in a different model focused on Alzheimer’s disease [34]. One of the natural components of oats is the polyphenol avenanthramide-C (Avn-C). Its regular use contributes to slowing cognitive decline and also plays a regulatory role in various pathological processes. Additionally, Avn-C supports the preservation of synaptic function. It also contributes to the reestablishment and maintenance of long-term potentiation (LTP). Cellular signaling pathways play a critical role in these effects. In particular, the activation of AMPK and the inhibition of GSK3β form the basis of the mechanism. These processes regulate amyloid accumulation and tau hyperphosphorylation. Avn-C also suppresses excessive neuronal apoptosis. Thus, caspase-3-mediated cell death is significantly reduced. The findings suggest that early and continuous use of Avn-C may be a potential approach for treating Alzheimer’s disease. Its multifaceted effects on neurodegenerative mechanisms stand out as one of the compound’s notable features in the literature.

The neuroprotective effects of polyphenols in PD have been demonstrated to be beneficial [18]. PD is a neurodegenerative disorder characterized by the degeneration of dopaminergic neurons and is typically associated with aging. Resveratrol (RES) activates various signaling pathways, especially SIRT1. This suppresses apoptosis and strengthens cellular defense mechanisms. Exosomes obtained from human neural stem cells (hNSC) pretreated with RES (RES-hNSC-Exos) have shown significant protective effects against neural damage. These exosomes preserve cell viability, support mitochondrial function, enhance antioxidant defense, and reduce inflammatory responses. Due to these properties, they are considered promising candidates for PD treatment. Another study on PD examined Chichoric Acid (CA), a polyphenolic compound derived from chicory and echinacea [82]. CA treatment reduced structural abnormalities in dopaminergic neurons in mice. It also alleviated motor symptoms and contributed to the suppression of glial cell activity. In addition, it has been reported to increase levels of dopamine (DA), brain-derived neurotrophic factor (BDNF), and 5-hydroxyindoleacetic acid (5-HT). It has also been shown that CA decreased interleukin-17 (IL-17) and interferon-gamma (IFN-γ) levels, while significantly increasing transforming growth factor-beta (TGF-β) levels in the spleen and colon [82]. Genome-level expression analyses have identified mechanisms that mediate the suppression of neuroinflammation by CA and appear to be linked to modulation of the brain-spleen and brain–gut axes in PD. In the spleen, the PPAR signaling pathway influences gene expression in processes such as epithelial–mesenchymal transition and TGF-β signaling. In the colon, important metabolic and immunological pathways were affected, including biochemical conversion processes of lipid derivatives, response to interferon-gamma (IFN-γ), activity of cytochrome P450 family biotransformation enzymes, and the PPAR signaling pathway. These findings strongly support the neuroprotective role of CA in PD, emphasizing its modulation of the peripheral immune system, particularly in the spleen and colon. The observations demonstrate that CA affects several mechanisms in these areas and underline its importance in the context of PD.

In summary, polyphenols have been evaluated in numerous studies as food-derived bioactive compounds for the prevention or treatment of neurodegenerative diseases, and the positive effects of polyphenol use on neurodegenerative protection have been emphasized. They effectively protect neuronal survival by penetrating the blood–brain barrier, scavenging ROS, enhancing autophagy, and regulating apoptotic signaling pathways [83].

Additionally, isocoumarins, one of the natural compounds structurally related to polyphenols, are notable for their neuroprotective activities [84]. Polyphenols play a protective role in neurodegenerative disease models. Isocoumarins, particularly 8-hydroxy-3-aryl isocoumarin derivatives, also increase synaptic plasticity and dendritic arborization by exhibiting an agonist effect on the Tropomyosin receptor kinase B (TrkB) receptor, offering therapeutic potential in conditions such as Alzheimer’s disease and depression. In conclusion, the capacity of polyphenols to modulate neurodegenerative processes is supported by the neuroprotective effects of structurally related isocoumarins via TrkB, offering therapeutic potential in conditions such as Alzheimer’s disease and depression.

### 4.2. Anti-Inflammatory and Antioxidant Support

In elderly individuals, the rates of tissue regeneration and cellular proliferation decrease [85]. The aging process renders physiological systems more vulnerable to health deterioration and potentially fatal outcomes. Furthermore, the senescence process triggers systemic inflammation, a key component of cellular aging. Inflammation that protects the body can take on a detrimental role with age. Chronic inflammation encourages oxidative stress by increasing the formation of ROS. ROS are generated through several mechanisms, including NADPH oxidase, xanthine oxidase, immune stimulation, and arachidonic acid metabolism; however, the primary source of ROS production is the mitochondria. The leading forms of ROS are superoxide anion (O_2_^•−^), hydrogen peroxide (H_2_O_2_), and hydroxyl radical (^•^OH). Low levels of ROS production play a role in modulating several physiological processes, including immune responses and apoptosis signals, but increased ROS levels can lead to various adverse outcomes. The relationship between ROS and inflammation is bidirectional, meaning that they can mutually exacerbate each other. ROS formation can lead to increased inflammation and damage to important cellular components, particularly DNA. Such processes form the basis of chronic diseases and functional disorders that arise with aging. Polyphenols, recognized for their significant antioxidant and anti-inflammatory properties, have been the subject of extensive research [1].

Polyphenols are notable for their capacity to prevent cell damage by combating free radicals. Isocoumarins, which share a similar structure with polyphenols, also exhibit comparable radical scavenging abilities [86]. For example, compound **1c**, a 3-aryl isocoumarin derivative, exhibited 53% radical scavenging activity, and this activity is linked to 5-lipoxygenase (5-LOX) inhibition. While polyphenols modulate inflammation, isocoumarins also exhibit similar anti-inflammatory effects by inhibiting the enzymes 5-LOX and microsomal prostaglandin E2 synthase 1 (mPGES1). Compound 1c has emerged as a dual inhibitor targeting these enzymes. These data demonstrate that polyphenols and structurally related isocomuarins can modulate oxidative stress and inflammation through multiple mechanisms.

Grape skins high in polyphenols were examined in one study [87]. The extracts demonstrated strong free radical scavenging activity along with a notable anti-inflammatory effect. These outcomes point to the high polyphenol content of grape peel as a major factor in counteracting oxidative stress and inflammation. In another study on grape polyphenols, the effects of compounds obtained from grape pomace (GP) on the intestinal antioxidant capacity and inflammatory response of pigs were investigated [88]. After GP administration, a significant decrease was observed in the expression of proinflammatory markers such as IL-1β, TNF-α, MHC-II, and NF-κB p65. However, Nrf2 plays a central role in antioxidant defense. Its level increased after treatment. The results obtained reveal that grape pomace strengthens cellular defense mechanisms by reducing inflammation and oxidative stress in the intestines. These findings also suggest that grape polyphenols may provide protective effects not only locally but also systemically.

Another study examined the anti-inflammatory and antioxidant properties of purified free polyphenols (P-RRTP-FP) and bound polyphenols (P-RRTP-BP) obtained from Rosa roxburghii Tratt Pomace (RRTP) [89]. The damage induced by inflammation is indicated by a decrease in mitochondrial membrane potential. After administering polyphenols, this potential was significantly stabilized. Additionally, evaluations using the DPPH and ABTS methods revealed that the free radical scavenging capacity was stronger compared to that of vitamin C.

The chronic liver disease known as NAFLD usually advances slowly and is strongly linked to insulin resistance [90]. It has been reported that Pandanus amaryllifolius Roxb. polyphenol extract (PAE) has anti-inflammatory and antioxidant properties that may help with NAFLD. In NAFLD mice fed a high-fat diet, PAE treatment substantially reduced oxidative damage in the liver tissue. Furthermore, both liver and serum levels showed a considerable rise in the activity of oxidative stress-related enzymes, including glutathione (GSH) and superoxide dismutase (SOD). By strengthening the antioxidant response, this increase helped to reduce lipid peroxidation and lessen liver damage.

In another study, a mouse model was used to examine the effects of polyphenols [91]. Proanthocyanidins (PA) are compounds that are commonly present in tea, wine, cereals, and legumes. On the other hand, amaranth and buckwheat leaves are used to make rutin. The purpose of this study was to compare the effects of these two polyphenols. This is one of the few studies that compared the modulatory effects of rutin and PA on gut microbiota, weight reduction, and fasting blood glucose (FBG) levels. Mice with a type 2 diabetes model induced by a high-fat diet (HFD) were divided into three groups: HFD + PA, HFD + Rutin, and a control group given physiological saline (HFD + SDSS). The PA-treated group showed inhibited adipocyte hypertrophy and a decreased inflammatory response. Histopathological analyses (H&E staining) and tissue measurements validated these findings. In addition, a significant decrease in liver steatosis and the density of lipid-containing vesicles was recorded. It has been reported that PA provides an anti-inflammatory effect by regulating various metabolic pathways in the gut microbiota. When evaluating the rutin supplement, rutin regulated the gut microbiota similarly to PA but with more effective results. It has been observed that the polyphenols used may have positive effects in alleviating the symptoms of T2DM mice.

Overall, polyphenols have a healing effect on various diseases and provide support to the body [92]. Their effectiveness is largely attributed to their antioxidant and anti-inflammatory characteristics.

### 4.3. Muscle and Bone Health

Sarcopenia and osteoporosis are musculoskeletal conditions of the muscle and skeletal system that have a serious impact on daily functioning by causing a decrease in motor competence [93]. Osteoporosis is a systemic skeletal disease marked by decreased bone and mineral density, leading to structural deterioration. Osteoporosis leads to an increased likelihood of fractures in bones that bear body weight, i.e., are subjected to mechanical loading. However, sarcopenia is a condition defined by a gradual decline in skeletal muscle mass, strength, and functional capacity, and there are no FDA-approved pharmacological treatments for sarcopenia. Natural compounds have therapeutic potential and are known to protect bone and muscle. There is evidence to support that polyphenols like oleuropein and oleocanthal found in the Mediterranean diet may improve muscle and bone health [93].

Further evidence for muscle-related effects comes from a study in which, using a combination of dutasteride with curcumin and resveratrol polyphenols in liposomal form, the potential for a positive effect against patients with Amyotrophic Lateral Sclerosis (ALS) was observed [94]. ALS is a neurodegenerative disease that involves motor dysfunction and musculoskeletal impairment. Due to this disease, affected individuals may fall, suffer fractures, and accelerate the development of sarcopenia. The study was conducted on elderly individuals with an average age of 56.74 years. After two months of intervention, a substantial increase in basal electrical activity of muscles was detected in the intervention group (IG). Compared to the control group (CG), it led to a more pronounced increase in basal electrical activity, especially in the right and left body hemibody muscles. The muscle activation of the individuals in the intervention group became more balanced, and the increase was more widespread in all muscles, especially in the upper muscle groups, which are the main reference point of the disease. In addition, the effect of the intervention on fasciculations (spontaneous and involuntary muscle contractions), an important symptom in the diagnosis of ALS, was examined. In the intervention group, there was an increase in the lowest (P10) and highest (P1) fasciculation peaks of the upper extremity muscles (right biceps brachii and left triceps brachii), and therefore, measurements of the total activity of the upper extremity muscles indicated a significant increase. In the control group, a significant increase in the frequency of fasciculations in the lower extremity muscles and loss of symmetry in the motor activation pattern were observed. These findings point to a reduction in spontaneous activation following the intervention. In elderly participants, polyphenols, especially curcumin and resveratrol, appeared to enhance muscle activity while also helping to slow down degenerative changes.

In another experiment, Kaempferia parviflora extract (KPE), which is rich in polyphenols, was given to mice, and it was found that KPE significantly increased the mice’s submaximal exercise capacity [95]. Following KPE treatment, mice showed an upregulation of antioxidant-related genes in skeletal muscle, accompanied by a rise in plasma antioxidant levels. KPE supplementation was associated with notable improvement in exercise performance parameters compared to the control group. Mice receiving supplementation had a 24.7% longer exercise duration and approximately 20% greater daily running distance compared to the control group.

Another study showed that polyphenols have beneficial effects on bone health [33]. RSV, found naturally in fruits like grapes and strawberries, has poor bioavailability. To increase its bioavailability, it was studied through a nanoparticle-loaded hyaluronic hydrogel system. RSV has demonstrated pro-osteogenic properties that promote bone formation. It enhanced the differentiation of osteoblasts, the cells responsible for bone formation, and showed a modulatory effect on elevated ALP (alkaline phosphatase) activity and calcium nodule formation. Furthermore, it inhibited the formation of osteoclasts, the cells responsible for bone resorption (RANKL-induced osteoclastogenesis). RSV has therapeutic potential in correcting bone formation disorders by modulating processes including osteoblast/osteoclast balance, bone regeneration, and inflammation control mechanisms.

Based on these findings, it is thought that polyphenols may play a potential supportive role in combating bone and muscle problems associated with aging.

### 4.4. Anti-Aging and Longevity-Promoting Effects of Polyphenols

Together with an improved quality of life, the trend of delaying the aging process, both individually and socially, has led to the search for innovative solutions to the biology of aging [25]. In this context, the regular use of various dietary polyphenol compounds is supported by preclinical and clinical findings that they have the potential to both prolong life and improve quality of life.

Quercetin (QUER) is a natural polyphenolic compound known for its life-extending and anti-aging effects [96]. In 2010, the FDA reported high-purity QUER as “Generally Recognized as Safe”. In current commercial applications, QUER is circulated in the food and supplement industry as a dietary supplement with claims of various health-promoting effects. Studies on yeast (Saccharomyces cerevisiae) have revealed that QUER supplementation at the beginning of chronological aging (diauxic shift) significantly extends chronological life span (CLS). As a result of antioxidant enrichment, a decrease in the levels of oxidative stress biomarkers, including superoxide anion (O_2_^−^) and MDA, was observed, which contributed to the increased CLS. It also played a regulatory role in carbon metabolism. It enhanced the catabolism of C2 by-products (ethanol and acetate) of yeast fermentation via Sir2-dependent phosphoenolpyruvate carboxykinase activity. In addition, it promoted a life-extending anabolic metabolism by increasing glycerol catabolism via the L-glycerol 3-phosphate (L-G3P) pathway. Through these signaling pathways, it triggered gluconeogenesis and, consequently, an increased content of trehalose, a disaccharide that accumulates in senescent cells and supports vital functions. A similar result was observed using a different experimental model [97]. The effects of QUER on the senescence of Drosophila intestinal stem cells (ISCs) were studied. Abnormal proliferation and differentiation of intestinal stem cells (ISC) can be observed in old Drosophila intestines. QUER supplementation prevents excessive ISC proliferation and the pathological increase in intestinal morphology leading to growth. This effect is associated with a remarkable reduction in stem and precursor cell populations (esg-GFP+, Dl+, pH3+ cells). Furthermore, it effectively maintains intestinal homeostasis and improves intestinal function, offering several benefits, including restoring intestinal acid-base balance, maintaining the integrity of the intestinal barrier, and accelerating the healing of damaged intestine. Experimental findings suggest that QUER supplementation may extend the lifespan of aged Drosophila model organisms.

Studies on curcumin and caffeine contribute to understanding the biological mechanisms involved in the aging process from different perspectives [98,99]. Curcumin, a phenolic compound, may indirectly contribute to extending lifespan by alleviating diabetic microangiopathies and its associated complications [98]. Mechanisms such as reducing oxidative stress, stimulating Nrf2 activation, and suppressing the inflammatory response mediate curcumin’s role in slowing age-related metabolic deterioration by protecting vascular functions. On the other hand, caffeine, a non-phenolic compound found in polyphenol-rich beverages, may similarly show positive effects related to lifespan by reducing cardiovascular mortality and all-cause mortality rates when consumed at moderate levels [99]. When these findings are considered together, it appears that other bioactive compounds found in the same sources, I addition to polyphenols, may also play complementary roles in aging and longevity.

Another study examined the effects of phlorizin (phloridzin) in *Caenorhabditis elegans* [100]. Phlorizin, a compound in the flavonoid class, is naturally present in apples before they ripen. The supplementation of phlorizin significantly prolonged both the mean and maximum lifespan in the *C. elegans* model system. The average lifespan increased by 18%. Genetic analysis indicated an anti-aging effect, potentially through mechanisms such as the DAF-16-dependent stress response and autophagy. In conclusion, numerous studies indicate that polyphenols may have anti-aging and life-prolonging effects through various mechanisms.

**Table 3:** Studies on Polyphenol Interventions in Elderly Individuals.

Polyphenol/ Compound	Dosage	Usage	Effects	References
Liposomal Curcumin, Resveratrol	In vivo	200 mg/day of curcuminoids and 75 mg/day of resveratrol for 2 months—Orally administered as an aqueous solution prepared in liposomal form, together with dutasteride	Increased acute muscle activation in ALS patients; an increase in total activation of upper extremity muscles was observed in fasciculations (involuntary muscle twitching)	[94]
Resveratrol	In vitro	15 µM—Loaded onto solid lipid nanoparticles and embedded in cross-linked hyaluronic acid hydrogel; drug delivery system	During the treatment of diabetes-related periodontitis, polyphenols exhibited anti-inflammatory, antioxidant, and bone-regenerating properties	[33]
Oleuropein and Polydatin	In vitro	3 nM oleuropein and 1.5 nM polydatin doses alone or in combination—Experimental condition	Improved muscle and bone metabolism; promoted the differentiation and development of osteoblasts (bone-forming cells) and myoblasts (muscle-forming cells)	[93]
Avenantramid-C	In vivo and in vitro models	6 mg/kg/day in mouse models. In in vitro experiments, 50 µM/day—Orally, in mouse models of Alzheimer’s disease (5xFAD and Tg2576); in vitro conditions in BV2 microglial cell models	Prevented or slowed progression of Alzheimer’s disease; preserved cognitive function, restored and sustained long-term potentiation	[34]
Resveratrol	In vitro	10 µg/mL—Experimental condition	Prevented neurotoxicity; increased cell viability; increased mitochondrial biogenesis and reduced production of reactive oxygen species	[18]
Phenolic compounds in Xianhu tea water extract	In vitro and in vivo	In *C. elegans* experiments, low concentration (L) was 1 mg/mL and high concentration (H) was 4 mg/mL—Xianhu tea powder obtained from tea leaves was inoculated onto agar plates with *E. coli* OP50 on *Caenorhabditis elegans* (*C. elegans*) nematodes	Strong antioxidant and anti-aging properties; *C. elegans* lifespan was extended, with a 23.50% and 21.07% longer lifespan observed compared to the control group	[101]
Glukozil Hesperidin	In vivo	1% G-Hes to drinking water for 4 weeks—Experimental condition	Prevented tubulointerstitial fibrosis and immune activation in diabetic nephropathy in mice	[11]
Raisin Polyphenol Extract	In vitro and in vivo	50, 100 and 200 µg/mL in cell studies; 100, 200 and 400 mg/kg/day—Experimental condition and oral gavage in mice	Demonstrated antioxidant and anti-aging effects in mice	[91]
Grape Pomace	In vivo	1 gGP/kg (E1), 5 gGP/kg (E2), 10 gGP/kg (E3) and 15 gGP/kg (E4) were given three times a day for 90 days—In feed, throughout the experiment	Improved growth performance in pigs; supported intestinal morphology; improved anti-inflammatory effect and antioxidant activity	[88]
*Pandanus amaryllifolius* Roxb. Polyphenol Extract	In vivo	100 mg/kg/day (low dose) and 200 mg/kg/day (high dose)—Orally	Alleviated non-alcoholic fatty liver disease in mice	[90]
Pomegranate Extract	In vitro	5 g pomegranate extract for in vitro digestion; 500 mg digested residue per tube for fermentation—Simulated in vitro human digestion (oral, gastric, intestinal) followed by in vitro fermentation with gut microbiota from healthy, obese, and celiac individuals	Modulated functionality of the gut microbiota	[102]
Polyphenols Extracted from *Rosa roxburghii* Tratt Pomace	In vitro	50 µg/mL and 100 µg/mL concentrations were used for cell studies—Experimental condition	Demonstrated anti-inflammatory effects in LPS-activated macrophages; demonstrated antioxidant effects with higher DPPH and ABTS radical scavenging capacity than vitamin C	[89]
*Houttuynia cordata*	In vitro	250 µg/mL and 500 µg/mL—Applied on NGM agar plates as dietary supplement	Decreased lipofuscin, a pigment associated with aging, demonstrating powerful antioxidant and anti-aging properties	[103]
Grape Seed Polyphenol Extract	In vivo	100 µg/mL—Experimental condition	Stroke caused by amyloid-beta improved significantly; the lifespan of model organisms increased	[81]
Black ginger (*Kaempferia parviflora*) extract	In vivo	8 weeks of dietary supplementation with a feed containing 3% (*w*/*w*)—Administered orally to mice by mixing it into their diet	Increase in submaximal endurance exercise capacity and daily voluntary wheel running distance	[95]
Oleuropein aglycone and Hydroxytyrosol	In vitro	12.5, 25, and 50 µM—Tested in vitro on neuronal and microglial cell models	Decreased release of proinflammatory cytokines (IL-6, IL-8, IP-10, RANTES, MIP1b); damage to neuronal cells was alleviated	[78]
Quercetin	In vitro	300 µM—Added to minimal medium/2% glucose culture at onset of chronological aging	Significantly increased chronological lifespan in yeast	[96]
Quercetin	In vivo	1, 10, 50, and 100 µM—In feed, throughout the experiment	Prevented excessive proliferation of intestinal stem cells (ISCs) in *Drosophila*, maintained intestinal homeostasis, and extended lifespan	[97]
Phlorizin	In vivo	1 µM, 100 µM, and 1000 µM—Experimental condition	Showed strong antioxidant and anti-aging properties, extended average and maximum lifespan	[100]
Chicoric Acid (CA)	In vivo	40 mg/kg/day for 12 days—Orally administered in an MPTP-induced mouse model	Prevented neuroinflammation and neurodegeneration	[82]

## 5. Discussion

In addition to the biological benefits of polyphenols, toxicity, interactions, and dose-dependent adverse effects must also be carefully considered. High-dose or long-term supplement use may lead to undesirable changes in liver function, body homeostasis, and gut microbiota. Furthermore, polyphenols have the potential to interact with medications and alter pharmacological. This situation may pose a clinical risk for elderly individuals taking multiple medications or populations with chronic diseases. Therefore, it is crucial to optimize dosages and establish safety limits that take into account factors such as age, gender, health status, and dietary patterns.

Although large-scale studies examining the relationship between nutrition and health support the link between polyphenol intake and chronic disease risk, confounding variables such as lifestyle, dietary patterns, socioeconomic status, and environmental factors complicate the interpretation of these results. Therefore, future studies require research designs that can more clearly distinguish the effects of variables such as age, gender, diet, and environmental factors.

In general, polyphenols have significant potential in terms of extending healthy life span, preventing age-related degenerative diseases, and maintaining quality of life. However, in order to utilize this potential in a safe, effective, and individualized manner, comprehensive clinical studies are needed that clearly define the risk-benefit balance and establish toxicity limits. A thorough understanding of the different biological effects of polyphenols according to life stages will shed light not only on reducing disease risk but also on multidimensional health gains such as maintaining cognitive function, metabolic balance, and psychological well-being. In this context, identifying age-specific polyphenol responses will significantly contribute to the development of healthy aging strategies and functional nutrition approaches in the future.

## 6. Conclusions

This review provides a comprehensive overview of the effects of polyphenols on different age groups, offering an up-to-date assessment of the age-specific biological functions of polyphenols. Polyphenols are a wide variety of bioactive molecules with properties including antioxidant, anti-inflammatory, anti-obesity, neuroprotective, and cardioprotective. They exert their effects through various biochemical mechanisms, including differential signaling mechanisms, epigenetic mechanisms, scavenging of free radicals, and modulation of gene expression. In the human body, the cellular, molecular, and systemic needs change with age. Because of these variations, it is crucial to examine the specific effects of polyphenols on each age group individually to create tailored health strategies. Polyphenols have different effects on individuals across various age groups. For example, the goals are to observe immune development in infancy, cognitive development in childhood, prevention of chronic diseases in adults, and neuroprotective effects that delay aging in the elderly. The differing effects of these compounds based on age could provide a scientific foundation for the development of functional food products in the future, considering the various age stages of individuals.

While the current literature primarily focuses on the general effects of polyphenols, comparative studies designed for the specific needs of each age group are quite limited. Although in vitro and animal studies have demonstrated the potential benefits of polyphenols, there is a lack of human-focused clinical data, particularly covering infancy and childhood. Therefore, there is a need for more controlled experimental studies to determine the effects of polyphenols on age, dose, method of use, and the physiological systems they influence. In conclusion, polyphenols possess diverse regulatory properties and offer considerable potential for creating individualized strategies that serve the specific needs of various age groups.

## Figures and Tables

**Table 1:** Studies on Polyphenol Interventions in Early Life and Childhood.

Polyphenol/Compound	Research Model	Dosage/Usage	Effects	References
Tea polyphenols	In vivo	0.00% (control), 0.02%, 0.04%, 0.08% of the compound—in feed, throughout the experiment	Improved antioxidant status; enhanced immune function; better intestinal health in young fish	[27]
Anthocyanins, ellagitannins, and quercetin	In vivo	0.8 mg/mL (prepared by dilution)—Drinking water	Increased sperm production, improved sperm morphology and fertility, enhanced plasma antioxidant capacity in father rats; reduced perinatal mortality rate in newborn rats	[26]
Hydroxytyrosol	In vivo	500 mg/kg (supplemented to basal diet for 28 days)—In feed, throughout the experiment	Alleviated intestinal damage caused by oxidative stress in piglets	[40]
Chlorogenic acid, protocatechuic acid, and eleutherosid	In vitro and ex vivo	100 µg (single dose)—Experimental condition	Inhibited human serum hyaluronidase activity by 76–86% in children with acute leukemia (average age 7)	[48]
Wild Blueberry	In vitro and in vivo	13.3 g/day for 4 weeks—Naturally obtained through the diet	Significant improvements in executive function and memory have been observed in healthy children (children aged 7–10)	[43]
Resveratrol and Resveratrol Butyrate Ester	In vivo	RBEL (Low Dose): 3.33 mg/kg/day; RBEH (High Dose): 6.67 mg/kg/day; 6 weeks during gestation and lactation in rats—In drinking water	Protected offspring from maternal factor-induced hypertension	[28]
Hydroxycinnamic acids, flavanols, flavanones, etc.	In vivo	5–20 mg/day—Naturally obtained through the diet	Improved lipid profile of breast milk	[31]
Cranberry (*Vaccinium* sp.)	In vivo	20 g/day for 21 days—Naturally obtained through the diet in breastfeeding mothers	Strengthened the antioxidant system of human milk; improved antioxidant status	[32]
Resveratrol	In vitro	60 µM and 120 µM—Experimental condition	Increased apoptosis without affecting normal cells in cell lines derived from osteosarcoma; reduced inflammation; stopped cell migration and enhanced the effect of chemotherapy	[44]
Alpha-mangostin, Nordihydroguaiaretic Acid	In vitro	α-mangostin: 10, 15, 20, 40 µM; NDGA: 25, 50, 75, 100, 200 µM; 24 h—Experimental condition	Cytostatic and cytotoxic effects on medulloblastoma have been observed	[49]
Resveratrol	In vivo	0.318% *w*/*v*/day after tooth brushing—Oral spray formulation; tested in children	Reduced gingivitis, improved saliva pH in children with plaque-induced gingivitis	[45]
Polyphenols from tea, cranberry, and raspberry	In vitro	0.78–6.25 mg/mL (10–20% brewed tea)—Experimental condition	Inhibited plaque bacteria and biofilm formation; reduced plaque adhesiveness	[50]
Resveratrol	In vitro	2.5, 5, 10, 20, 40 µM—Encapsulated in TPGS microspheres in human keratinocyte HaCaT cells	Potential positive effects of combining RES and TPGS in the treatment of chronic liver disease have been observed	[46]
Hydroalcoholic extract of *P. oceanica* leaves	In vitro	3 µg/mL—Using nanocarrier systems	Migration of SH-SY5Y cell lines was significantly reduced in neuroblastoma	[51]

**Table 2:** Studies on Polyphenol Interventions in Adults.

Polyphenol/ Compound	Research Model	Dosage/Usage	Effects	References
Polyphenols from Chokeberry (*Aronia melanocarpa*)	In vivo	90 mg/day (capsule) or 300 mL/day (fruit juice)—Extracts/powders in capsules or as fruit juice	Potential reduction in cholesterol and LDL-C; potential reduction in systolic blood pressure (with high doses of anthocyanin); potential increase in fasting blood sugar in people under 50 years of age	[59]
Resveratrol	In vivo	Dose of 100 mg/kg/day for dogs for 35 days; doses of 100, 200, and 300 mg/kg/day for mice for 35 days—In dogs, in starch capsule added to basal diet; in mice, via oral gavage	Improved stress-related behaviors in dogs and mice	[70]
Pomegranate Extract	In vivo	740 mg/day for 12 weeks—In capsule form	Reduced inflammatory markers (IL-6 and IL-1β) and systolic blood pressure	[71]
Cocoa flavanols (e.g., (−)-epicatechin, (+)-catechin, and proanthocyanidins)	In vivo	25 gr/day of dark chocolate with a cocoa content of 85% or 88%, five times a week—As nutritional support 40 min before training or competition	Reduced muscle soreness and improved physical performance in young elite soccer players	[61]
Coffee and Olive Pomace	In vivo	Coffee grounds containing 10% olive pomace by weight—In feed, throughout the experiment	Led to more exploration and less rest time in mice; this increased fluid consumption in mice by 90%	[72]
Almond Hull Extract	In vitro and in vivo models	0.0625–1 mg/mL (lipase inhibition); 0.1–0.4 mg/mL (cell experiments); 0.1 mg/mL (in vivo studies)—Experimental condition	Inhibited pancreatic lipase activity; reduced intracellular triglyceride accumulation; increased cellular antioxidant capacity; modulated PI3K-AKT signaling; improved insulin resistance	[65]
Mulberry (*Morus alba*) branches and leaves extract	In vivo	500 mg/kg/day (low dose) or 1000 mg/kg/day (high dose) for 8 weeks—Oral gavage	Effectively improved obesity-related metabolic disorders in obese mice	[66]
Glukozil Hesperidin	In vivo	239 mg G-HES daily on average over 4 weeks—Orally, 1% G-HES added to drinking water	Prevented tubulointerstitial fibrosis and immune activation in rat model	[11]
Black Chokeberry (*Aronia melanocarpa*)	Ex vivo	1–500 µg/mL; 12 h—Dissected rat aortic rings treated with Ang II, LPS, or high glucose	Reduced inflammation and oxidative stress; modulated vascular responses	[60]
Thai Mulberry (*Morus alba* L.)	In vivo	100 g/day of CMD for 6 weeks—2 servings per day as a beverage	Reduced systolic and diastolic blood pressure, mean arterial pressure, and triglyceride levels; demonstrated anti-inflammatory effects by stabilizing fasting plasma glucose levels and reducing C-reactive protein levels in obese individuals	[67]
Coffee, tea, and red wine	In vivo	For up to 1 cup of coffee or tea per day and up to 1 glass of red wine per day (moderate consumption) for 6 weeks—Naturally obtained through the diet	Reduced perceived stress and depressive symptoms in adults	[69]
Resveratrol, Kaempferol, and Proanthocyanidins	In vitro	30 µM—Experimental condition	Increased anti-inflammatory M2 macrophage activation in mouse J774 and human U937 macrophage cells	[73]
Malvidin-3-*O*-glucoside	In vivo	12.5 mg/kg/day for 6 weeks—Oral gavage, mixed with drinking water	Reduced stress-related anxiety and depression-like behaviors in mice; reduced inflammation (particularly IL-1β protein levels)	[36]
Lingonberry (*Vaccinium vitis-idaea* L.)	In vivo	A high-fat diet supplemented with 20% by weight of air-dried cranberry powder for 6 weeks—Administered to mice in addition to their high-fat diet	Reduced weight gain in the body and liver; prevented adverse changes in the liver in mice	[19]

## Data Availability

No new data were created or analyzed in this study. Data sharing is not applicable to this article.
